# Selective screening for lysosomal storage disorders in a large cohort of minorities of African descent shows high prevalence rates and novel variants

**DOI:** 10.1002/jmd2.12201

**Published:** 2021-01-27

**Authors:** Renuka Pudi Limgala, Vyacheslav Furtak, Margarita M. Ivanova, Erk Changsila, Floyd Wilks, Marie N. Fidelia‐Lambert, Ozlem Goker‐Alpan, Marjorie C. Gondré‐Lewis

**Affiliations:** ^1^ Lysosomal and Rare Disorders Research and Treatment Center Fairfax Virginia USA; ^2^ Developmental Neuropsychopharmacology Laboratory, Department of Anatomy Howard University College of Medicine Washington District of Columbia USA; ^3^ Department of Pathology Howard University Hospital Washington District of Columbia USA

**Keywords:** African‐Americans, Fabry disease, Gaucher disease, large‐scale screening, lysosomal storage disorders, Pompe disease

## Abstract

Population studies point to regional and ethnicity‐specific differences in genetic predisposition for some lysosomal storage disorders (LSDs). The aim of the study was to determine the prevalence of the three treatable forms of lysosomal storage disorders (Gaucher disease [GD], Pompe disease [PD], and Fabry disease [FD]) in a cohort of mostly urban‐dwelling individuals of African ancestry, a previously unknown genetic landscape for LSDs. Large‐scale selective multistep biochemical and genetic screening was performed in patients seeking healthcare for various health concerns. Fluorimetric enzyme assays for GD, PD, and FD were performed on dried blood spots. Targeted gene sequencing was performed on samples that showed significantly lower enzyme activities (<10% of control mean) after two tiers of enzymatic screening. A total of 5287 unique samples representing a cross section of patients who visited Howard University Hospital and College of Medicine from 2015 to 2017 were included in the study. Study samples were obtained from a population where ~90% reported as African‐American, ~5% Hispanic, and <5% Caucasian or other. Regarding GD, three subjects had either homozygous or heterozygous mutations in the *GBA* gene. As to PD, eight subjects were either homozygous or compound heterozygous for *GAA* mutations, including three novel mutations: (a) c.472 A > G; p.T158A, (b) c.503G > T; p.R168L, (c) c.1985del. Regarding FD, two subjects had pathogenic *GLA* mutations, and four had single nucleotide polymorphisms in the 5'UTR, previously implicated in modulating gene expression. The findings highlight a higher incidence of abnormal enzyme levels and pathogenic mutations in the target population reflecting ancestry‐based specific genotype and phenotype variations.

**Synopsis**

Selective screening for lysosomal diseases in minority groups in the United States shows higher prevalence rates and novel variants.

## 1 INTRODUCTION

Lysosomal storage disorders (LSDs) are a group of about 50 inherited metabolic disorders resulting usually from mutations in the genes encoding lysosomal enzymes or enzymatic cofactors in the substrate degradation pathways. The advent of enzyme replacement therapy followed by the development of substrate reduction and pharmacological chaperone therapies enabled different treatment alternatives for some of the common LSDs, including Gaucher (GD), Pompe (PD), and Fabry (FD) diseases (OMIM # 230800, 232 300, and 301 500, respectively). As a result of treatment access, the natural history of these disorders has significantly changed, with a marked decrease in morbidity and mortality.^1^, ^2^ However, it has been noted that delaying the initiation of therapy results in disease progression and complications, which then persist even after therapy has commenced.^3^ Hence, awareness and early diagnosis of these LSDs followed by prompt treatment enables alleviating the symptoms and stopping the progression of the disease. The importance of early diagnosis has been recognized, and screening for these LSDs is now included in newborn screening pilot initiatives in some states of the United States as well as other countries.^4^, ^5^, ^6^, ^7^, ^8^ Nonetheless, adults with known clinical symptoms and comorbidities associated with these LSDs must also be informed about incidences to seek proper care.

All three of these LSDs have juvenile and/or adult presentations with attenuated phenotypes.^9^, ^10^ While infantile forms of GD and PD are easily recognizable with a unique and universal phenotype, a less severe, later disease onset in GD and PD poses significant diagnostic challenges. Patients carrying the less severe pathogenic variants may start to develop the symptoms at later ages in life and may initially present with common symptoms such as pain and fatigue. FD, which follows an X‐linked recessive inheritance pattern, is regarded as an adult onset disorder. Unless identified through a family screening, FD patients are often diagnosed due to advanced disease complications such as end‐stage renal disease, cardiomyopathy, and stroke. Varying disease onset and variable organ involvement in affected females further complicates the diagnostic odyssey.

Whereas individual LSDs are categorized as rare orphan disorders, LSDs as a group are relatively common and affect a considerable number in the general population with a combined incidence of 1:5000 to 1:8000.^11^, ^12^ Epidemiological studies undertaken in various countries estimate the GD incidence rate as ranging from 1 in 40 000 to 60 000,^13^ late‐onset Pompe disease (LOPD) as 1 in 40 000^14^ and FD as 1 in 50 000 males.^15^, ^16^ However, there is no clear information available on the prevalence/incidence rates of these LSDs in different ethnic groups or even overall in the US population. Certain LSDs are known to affect people across various racial or ethnic groups, and indeed, population studies point to regional and ethnicity differences which must be considered in planning for healthcare service and delivery.^17^ When ethnicity is considered, a handful of recent studies identified novel genetic mutations in LOPD in ethnic Australians,^17^ Chinese,^18^ Japanese,^19^ and Brazilians.^20^ The Brazilian study was heterogeneous in ethnicity, including individuals of Caucasian, African‐, and Asian ancestry. For GD, a study including seven African‐Americans found significant genotypic heterogeneity and pathogenic recombinations within the *GBA* gene.^21^ The prevalence of these or any of the LSDs is yet to be determined in minority/ethnic groups residing in the Unites States.

In the current study, the incidence of three LSDs was investigated in a cohort of mostly urban‐dwelling African‐Americans, compared to previous work primarily in Caucasians. These LSDs were chosen for two reasons, (a) several treatments are available, with more novel therapies in the pipeline, and (b) these LSDs have adult onset forms of the disease. In this prospective screening study, leftover blood samples were screened by enzymatic assays and targeted gene sequencing to determine the prevalence of GD, PD, and FD in individuals of African ancestry. Our results show a higher than expected incidence of these rare LSDs in the study cohort of 5287 samples; the incidence for GD was 1:1800, 1:700 for PD, and 1:900 for FD when assayed for enzyme activity followed by exon sequencing. Furthermore, at least three novel variants were identified for PD.

## 2 METHODS

### 2.1 Study design

The study was reviewed and approved by Howard University Institutional Review Board (IRB‐14‐MED‐09) and the Western Institutional Review Board (NCT02120235) and was conducted in accordance with the ethical principles described in Declaration of Helsinki of 1975. Samples (n = 5287) consisted of the randomized collection of left‐over blood samples during a period spanning 2015 to 2017, at Howard University Hospital, Washington, DC. Blood samples used as positive controls for GD (n = 25), obligate carriers of GD (n = 25), PD (n = 12), FD (n = 25), and healthy controls (n = 60) were obtained at the Lysosomal and Rare Disorders Research and Treatment Center, Fairfax, VA.

### 2.2 Preparation of dried blood spots

Leftover blood samples that were collected in K2 EDTA blood collection tubes were used to prepare dried blood spots (DBS) within 24 hours of blood draw. The positive and negative control samples collected at LDRTC in a similar manner were also used within 24 hours of blood draw. About 60 μL and 30 μL of blood sample from each tube was spotted onto Whatman 903 protein saver cards and FTA cards (GE Healthcare, Wauwatosa, WI) respectively. The cards were air‐dried overnight at room temperature and protein saver cards were stored at −20° C, while FTA cards were stored at room temperature until further use. The samples were stripped of personal identifiers and given unique identification codes to match only with the age, sex, and race of the blood donor.

### 2.3 Enzymatic analysis in DBS samples

Fluorimetric enzyme assays for lysosomal enzymes β‐glucocerebrosidase (β‐glu, EC 3.2.1.45), acid α‐glucosidase (α‐glu, EC 3.2. 1.20) and α‐galactosidase (α‐gal, EC 3.2. 1.22) were performed following the original method developed by Chamoles et al,^22^ with some modifications to fit the 384 well plates. Briefly, two 1 mm punches from DBS were added to 80 μL of the extraction buffer in 96‐well extraction plates for 1 hour. About 10 μL of the extracted samples were added to 20 μL of substrate buffer per well in triplicates. After 20 hours of incubation at 37°C, 50 μL of 0.5 M NaOH‐Glycine stop buffer was added per well, and the fluorescence was measured at 355 nm (excitation) and 460 nm (emission) on Filtermax F5 (Molecular devices, San Jose, CA). Enzyme activities were expressed as nanomole hydrolyzed substrate per hour per millilitre of blood.

### 2.4 Statistical analysis

Statistical analysis was performed using GraphPad Prism software (GraphPad Software, Inc., La Jolla, CA). Enzyme values were plotted, and graphs were generated as Box & Whisker (Tukey) plots. Data are expressed as average values ± the SE of the mean (SEM).

### 2.5 Genotyping

The samples were genotyped using 1 mm punches from FTA cards that were added to the PCR mix in a 50 μL PCR reaction. Amplifications of *GBA* (gene ID: 2629), *GLA* (gene ID: 2717) and *GAA* (gene ID: 2548) were performed in seven, four, and five reactions, respectively, using the primers listed in Table S1. Primers for *GBA* were designed to selectively amplify the *GBA* gene and not the pseudogene sequence. PCR amplification was optimized by using Takara PrimeSTAR GXL Polymerase (Clontech, Mountain View, CA). The DNA was denatured for 30 seconds at 94°C followed by 40 cycles of denaturation at 98°C for 10 seconds, annealing at 59°C for 50 seconds, and elongation at 68°C for 4 minutes. Amplified DNA was purified with DNA Clean and Concentrator kit (Zymo research, Irvine, CA), followed by fragmentation and preparation of libraries using NEBNext Fast DNA Fragmentation & Library Prep Set for Ion Torrent (New England BioLabs, Ipswich, MA). Concentration of libraries was measured with quantitative PCR using the universal library quantification kit for Ion Torrent (Thermo Fisher Scientific, Waltham, MA). Samples were sequenced on the Ion Torrent Personal Genome Machine. In‐house scripts written on C++ and publicly available sequence aligner programs (Bowtie, STAR) were used to analyze the generated DNA‐sequencing data. For the analysis of DNA‐sequencing, sequence reads were aligned against *GBA*, *GAA* or *GLA* genes sequence and single nucleotide profile was further analyzed against NCBI mutation database and Human Genome Mutation Database (HGMD) for GD, PD, and FD. Data were searched for pathogenic mutations.

## 3 RESULTS

**FIGURE 1:**
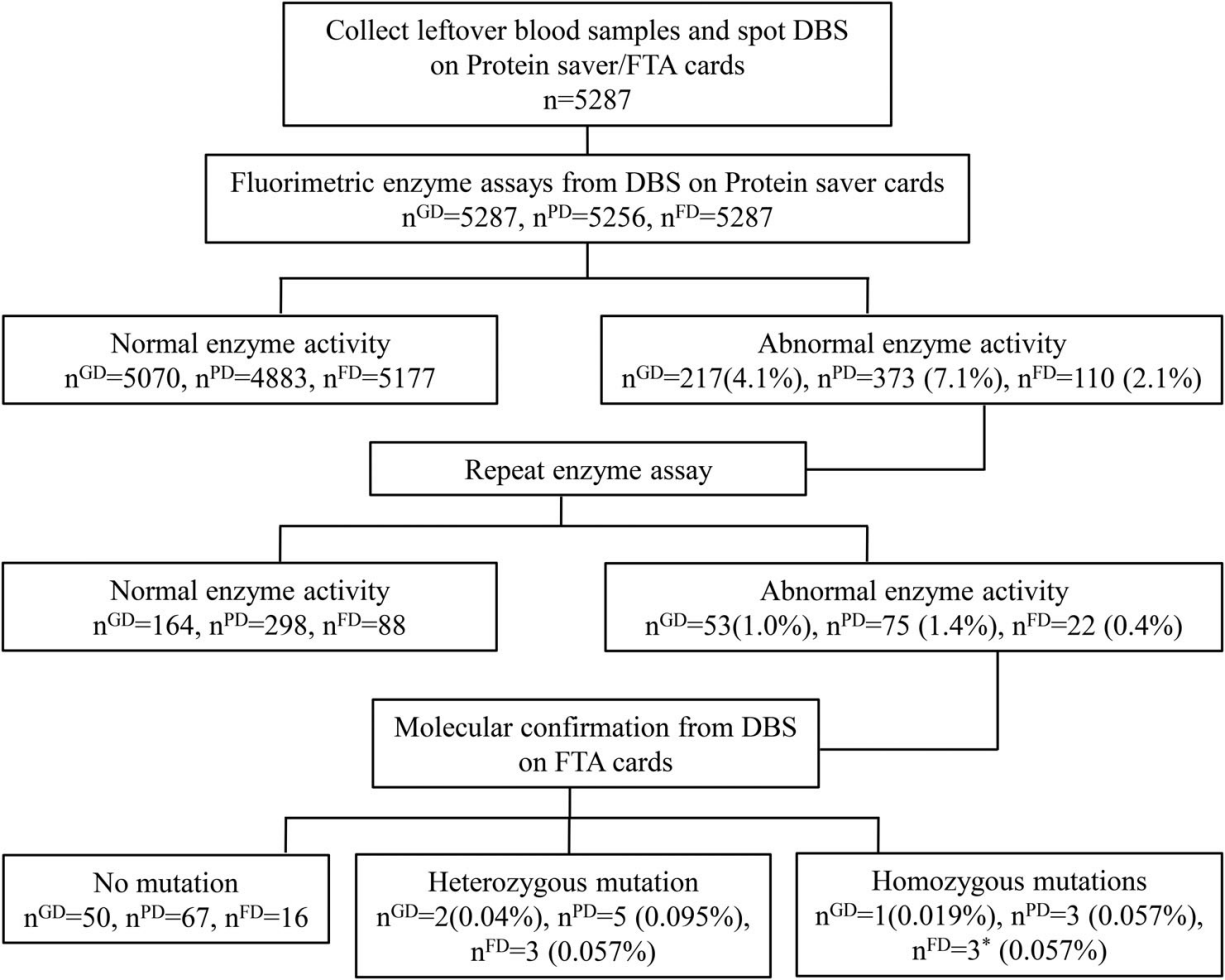
Description of the screening protocol. For each sample, blood was spotted onto Protein Saver and Flinders Technology Associates (FTA) cards and allowed to dry. Dried blood spots (DBS) from ProteinSaver cards underwent two tiers of enzyme assays. For samples with abnormal enzyme activity, DBS from the corresponding FTA cards were used for molecular analysis. Number of samples and percentage of total samples is indicated at each step. n^GD^, number of samples screened for Gaucher disease; n^PD^, number of samples screened for Pompe disease; n^FD^, number of samples screened for Fabry disease; * Hemizygous

**FIGURE 2:**
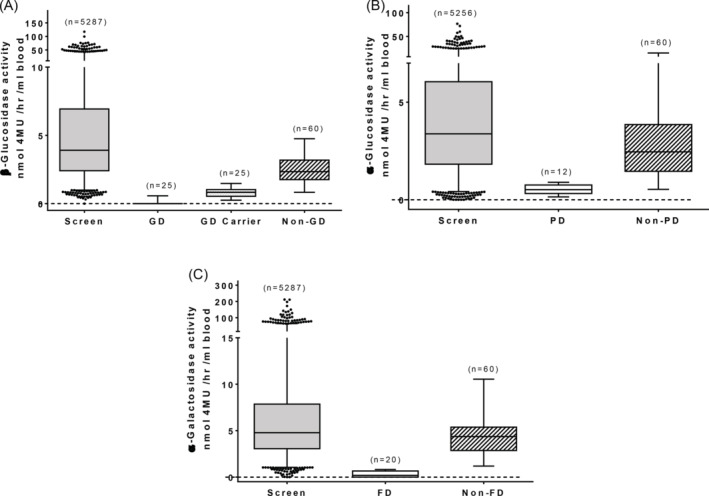
Results of enzyme assay screening. Fluorimetric enzyme assays were performed on screen and control cohorts and results were plotted as nmol of 4MU released/hour/mL of blood. The number of samples in each cohort is indicated. 4MU, 4‐methylumbelliferone; GD, Gaucher disease; PD, Pompe disease; FD, Fabry disease

The blood samples analyzed reflected 5287 urban‐dwelling, mostly African ancestry patients seeking clinical care for various health concerns and of these, 90% reported as African‐American, 5% Hispanic and 5% Caucasian or other. The age range of patients was 15 to over 100 years, with almost equal distribution of males to females. The experimental flow is represented in Figure 1. The enzyme activities were compared to previously confirmed GD samples (n = 25), obligate GD carriers (n = 25), PD samples (n = 12), FD samples (n = 25), and normal controls (n = 60). The enzyme activity results are shown in Figure 2 and summarized in Table 1.

**TABLE 1:** Statistical analysis of enzyme activities. Fluorimetric enzyme assays were performed on screen and control cohorts and statistical analyses for each enzyme activity are summarized

	# of samples	Minimum	Maximum	Median	1% percentile	99% percentile	Mean	SD	SEM
β‐Glucocerebrosidase									
Screen	5287	0	117	3.9	0.99	43	6.3	7.9	0.11
GD	25	0	0.57	0	0	0.57	0.04	0.13	0.025
GD carrier	25	0.25	1.5	0.83	0.25	1.5	0.81	0.32	0.064
Non‐GD	60	0.83	4.8	2.3	0.83	4.8	2.4	0.94	0.12
α‐Glucosidase									
Screen	5256	0	77	3.4	0.42	24	4.9	5.2	0.071
PD	12	0.14	0.89	0.52	0.14	0.89	0.52	0.24	0.069
Non‐PD	60	0.53	15	2.5	0.53	15	3.3	2.9	0.37
α‐Galactosidase									
Screen	5287	0	212	4.8	1.1	63	7.8	12	0.17
FD	20	0	0.82	0.19	0	0.82	0.31	0.32	0.072
Non‐FD	60	1.2	11	4.4	1.2	11	4.4	2.2	0.28

*Note*: All enzyme activities were indicated as nmol of 4MU released/hour/mL of blood.

Abbreviations: SD, Standard deviation; SEM, standard error of mean.

For all three LSDs, the average and range of enzymatic values for this mostly African‐American population are different from the LSD‐negative control reference group (Figure 2). β‐Glu activity ranged from 0 to 117 nmol/hour with a mean value of 6.3 ± 0.11 compared to a range of 0.83 to 4.8 and a mean of 2.4 ± 0.12 for non‐GD Caucasian controls (Figure 2A, Table 1). In the screen for PD, α‐glu activity ranged from 0 to 77 with a mean value of 4.9 ± 0.07, whereas control non‐PD Caucasians had an activity range of 0.53 to 15 and a mean of 3.3 ± 0.37 (Figure 2B, Table 1). Finally, α‐gal activity ranged from 0 to 212 with a mean value of 7.8 ± 0.17 compared to an activity range of 1.2 to 11 and a mean of 4.4 ± 0.28 for non‐FD Caucasian controls (Figure 2C, Table 1). Strikingly, 182 (3.4%) subjects had ~10 times higher β‐glu activity as compared to the mean enzyme activity of controls. Similarly, 26 (0.5%) and 109 (2.1%) subjects exhibited ~10 times α‐glu and α‐gal activities, respectively, compared to mean control enzyme activities. This was a highly unexpected finding. The higher values from different enzymes do not correlate within the same samples ruling out discrepancies in sample preparation.

To identify GD patients and accommodate possible GD carriers from the first tier of screening, a cut‐off of β‐glu activity was set at less than 1.0 nmol of substrate released/mL of blood/hour. After the first tier of screening, 217 (4.1%) samples presented with activity below the cutoff and the β‐glu assay on these samples was repeated in 96‐well plates for second tier testing. Of these, 53 (1.0%) samples showed enzyme activity lower than 1 nmol of substrate released/mL of blood/hour. FTA cards corresponding to the 53 samples were used to sequence the *GBA* gene (Figure 1). One subject showed a homozygous pathogenic mutation, c.476 G > A in exon 6/p.R159Q and two samples showed heterozygous mutations, a benign c.38 A > G in exon 3/p.K13R and pathogenic c1448 T > C and c.1483 G > C in exon 11/ p.L444P/p.A456P indicating GD carrier status (Table 2). No pathogenic mutations were found in other samples.

**TABLE 2:** Mutations and variants found for *GBA*, *GAA*, and *GLA* genes

	Enzyme activity (nmol 4MU/hr/mL)	cDNA	Protein	Zygosity	Variant
Gene: *GBA*					
1	0.51	1448 T > C	L444P	Hetero	Pathogenic
		1483G > C	A456P	Hetero	Benign
2	0.52	476 G > A	R159Q	Homo	Pathogenic
3	1.8	38A > G	K13R	Hetero	Benign
Gene: *GAA*					
1	0.00	1726G > A	G576S	Hetero	Benign/likely benign
		2065G > A	E689K	Hetero	Benign/likely benign
		596A > G	H199R	Homo	Benign
		668 G > A	R223H	Homo	Benign
2	0.00	596A > G	H199R	Homo	Benign
		668G > A	R223H	Homo	Benign
3	0.00	596A > G	H199R	Hetero	Benign
		668 G > A	R223H	Hetero	Benign
4	0.00	596A > G	H199R	Hetero	Benign
		668 G > A	R223H	Hetero	Benign
5	0.00	2560C > T	R854^a^	Hetero	Pathogenic
		2446G > A	V816I	Hetero	Benign
6	0.03	2446G > A	V816I	Hetero	Benign
		1985del		Hetero	Novel
7	0.05	2560C > T	R854^a^	Hetero	Pathogenic
		596A > G	H199R	Homo	Benign
		668 G > A	R223H	Homo	Benign
8	0.05	596A > G	H199R	Hetero	Benign
		668 G > A	R223H	Hetero	Benign
		472 A > G	T158A	Hetero	Novel
		503G > T	R168L	Hetero	Novel
Gene: *GLA*					
1	0.00	335 G > A	R112H	Hemi	Pathogenic/likely pathogenic
2	0.91	427 G > A	A143T	Hemi	Conflicting interpretations of pathogenicity
3	0.03	−10 C > T	5' UTR^b^	Hemi	
4	0.28	5049 C > T	5' UTR^b^	Hetero	
5	0.44	−10 C > T	5' UTR^b^	Hetero	
6	0.47	−10 C > T	5' UTR^b^	Hetero	

*Note*: All enzyme activities were indicated as nmol of 4MU released/hour/ml of blood.

^a^ Premature stop codon.

^b^ Mutations found in 5'UTR for *GLA* gene.

For α‐glu and α‐gal activities, screens for PD and FD, respectively, the cutoff for second tier re‐analysis was set at 10% of the mean enzyme activities obtained from the screen samples. After two tiers of enzymatic screening, 75 (1.4%) and 22 (0.42%) samples with low enzyme activities were identified. The corresponding FTA cards were sequenced for *GAA* and *GLA* genes. Results from *GAA* sequencing showed a total of eight samples with two or more mutations. Two subjects had premature stop codon mutations (c.2560C > T/p.R854*) while one showed a deletion mutation (c.1985 del). Two subjects with undetectable or low α‐Gal activity showed hemizygous pathogenic mutations, c.335 G > A/p.R112H and c.427 G > A/p.A143T, respectively. Four samples showed variants in 5'UTR of *GLA* gene (Table 2).

## 4 DISCUSSION

There are extensive studies accounting for epidemiological, clinical, biochemical, genetic, and therapeutic inquiries of LSDs in Caucasian populations in the United States and internationally. A recent search of Pubmed in December 2020 for “African‐American” and “Gaucher,” or “Pompe,” or “Fabry” yielded 13, 6, and 5 publications, respectively. Many of these articles were single case reports. Therefore, the findings in this study are opportune and much needed for more inclusive healthcare delivery regarding these rare disorders. Here, we report a higher than expected incidence of GD, PD, and FD in a mostly Black patient population. We further report three novel polymorphisms in the *GAA* gene impacted in PD.

GD and PD are collectively, two of the most common LSDs inherited in an autosomal recessive manner^11^ while FD is an X‐linked disorder.^15^ Due to the ambiguous presentations, patients are often not treated for the LSD, but rather, they are treated symptomatically without long‐term benefit. This invariably causes significant delays in diagnosis, often up to 10‐20 years with at least three different specialist visits. African‐Americans with high incidences of cardiovascular disease, lung scarring, and chronic kidney disease requiring dialysis may also possess hormone and metabolic imbalances; conditions that correspond to GD, PD, and FD clinical presentations.^16^, ^23^, ^24^, ^25^ People of African ancestry are known to be genetically heterogeneous and have traditionally been excluded in large research cohorts when investigating various diseases^20^, ^21^, ^26^ and thus in the age of precision medicine, if not studied, entire groups of individuals will be excluded from targeted treatment development. Because of the heterogeneity observed in people of African ancestry, their inclusion in studies such as this will lead to the identification of novel allelic polymorphisms that expand our current knowledge.

In addition to specific mutations in the various genes associated with GD, PD, and FD in this unique population, our findings suggest a never before reported increased biochemical activity for β‐glu, α‐gal, and α‐glu that bypasses by up to 10‐fold the highest expected range in “control” samples. At present, the significance of increased enzymatic activity in this or other populations is not known. In addition, even when enzymatic activity was zero or very low, this did not always correspond to a mutation following gene sequencing. Therefore, it is possible that some low enzymatic values correspond to other as of yet unidentified metabolic problems.

Recognizing the importance of screening for common forms of LSDs, they are included in many pilot newborn screening (NBS) programs.^4^, ^5^, ^27^ In the next few years, the data compiled from NBS will provide a better understanding of the LSD incidence and prevalence. Nonetheless, NBS data might not reveal underlying differences in incidence rates based on race/ethnicity and may result in the underestimation of LSD prevalence in specific ethnic/racial groups if evaluating them with the general population. With all its advantages, it should be noted that NBS is an asymptomatic form of screening which aims to identify affected individuals deficient in specific enzyme activity. This provides valuable information on birth prevalence, but there is still an unmet need for population screening and for identifying patients with reversible tissue damage. Diagnostic challenges are further exacerbated in minority populations due to the (a) the commonness of the wide variety of symptoms associated with LSDs^16^, ^23^, ^24^, ^25^ and (b) existing health care disparities in minority communities.

Since GD, PD, and FD can be managed therapeutically,^27^ it is vital to identify and treat patients early in order to avoid organ damage. According to the 2014 census studies by united states census bureau, minorities (including African‐American, Hispanic and Asians) make up for more than 35% of the US population, which is expected to increase further in the next few years. The incidence of these LSDs could be very different in specific minority groups, for example, in the African‐American population, which is as yet not tested. Moreover, there is no national or international data on GD, PD, and FD prevalence published on African‐Americans. Our study is the first of its kind to investigate such a large African‐American cohort for LSDs. In this study, we found a 3:5287 individuals had either homozygous or compound heterozygous mutation in the *GBA* gene (GD), 8:5256 in the *GAA* gene (PD), and 3:5287 in the *GLA* gene (FD). These high rates could be because this cohort includes sick individuals seeking inpatient and outpatient healthcare. Nonetheless, this was not an LSD clinic, and such findings have not been previously reported for other groups, including Caucasians at other healthcare settings.

It is highly relevant to US health care system to be aware of any racial influences in the prevalence rates in order to properly focus on clinical care needs. According to the 2017 Census Bureau data, the population of the District of Columbia (DC), was 47.1% Black or African‐American, 45.1% White (36.8% non‐Hispanic White), 4.3% Asian, 0.6% American Indian or Alaska Native, and 0.1% Native Hawaiian or Other Pacific Islander. Individuals from two or more races made up 2.7% of the population. Hispanics of any race made up 11.0% of the District's population.^28^ Howard University Hospital is a minority serving institution, with patients being 90% of African ancestry. Prior to launching this study, we conducted a preliminary review of LSD diagnoses at Howard University Hospital from January 1, 2010 through December 31, 2012. This study validated the possibility that LSDs were diagnosed by some physicians serving this population, likely without the benefit of genetics, and further supported the need for screening focused on minority groups. During the 2‐year period, 11 patients were diagnosed with a glycogen storage disease, such as PD. All 11of those diagnosed were African‐American. At the same time, four more patients were diagnosed with lipid storage diseases like FD and GD. Three out of those four patients were African‐American, while one patient was Caucasian.

The results from the large‐scale undertaking in the current study provide much needed population data of GD, PD, and FD prevalence in African‐Americans. One of the limitations of the present study is that the study population was already under clinical care for various reasons and thus the results may not represent the true prevalence rates in the general non‐hospital African‐American population. Another limitation is the inability to assign clinical symptoms and phenotypic correlation with the enzymatic levels and genetic variants. Nonetheless, given the fact that the diagnostic assays for these LSDs can be performed with relatively low cost and results can be confirmed using targeted single‐gene sequencing, it would be advisable for the physicians responsible for the clinical care of these patients to be aware that these LSDs, which are considered rare diseases in the general population, may be more common in people of African ancestry and other minority groups. Moreover, a higher incidence of LSDs observed in African‐American predominant minority groups provides another example for current existing disparities in health care. Underrepresentation of minority groups for both participation in clinical research trials and seeking specific medical care further hamper the clinical practice specifically addressing the medical needs of patients of African descent.

## CONFLICT OF INTEREST

Ozlem Goker‐Alpan acts as a consultant and has received speaker honorarium from Shire (now part of Takeda Pharmaceutical Company ltd.) and Pfizer. Renuka Pudi Limgala, Vyacheslav Furtak, Margarita M. Ivanova, Erk Changsila, Floyd Wilks, Marie N. Fidelia‐Lambert, and Marjorie C. Gondré‐Lewis have no conflicts of interests to disclose. The reporting of results of the study and decision to publish this manuscript are independent of the above conflicts.

## AUTHORS CONTRIBUTION

**Ozlem Goker‐Alpan and Marjorie C. Gondré‐Lewis**: Study concept and design. **Renuka Pudi Limgala, Vyacheslav Furtak, Margarita M. Ivanova, Erk Changsila, Floyd Wilks, and Marie N. Fidelia‐Lambert**: Acquisition of data. **Renuka Pudi Limgala and Marjorie C. Gondré‐Lewis**: Data curation. **Renuka Pudi Limgala, Vyacheslav Furtak, Margarita M. Ivanova, Ozlem Goker‐Alpan, and Marjorie C. Gondré‐Lewis**: Analysis and interpretation of data. **Renuka Pudi Limgala, Ozlem Goker‐Alpan, and Marjorie C. Gondré‐Lewis**: Drafting of the manuscript. **Vyacheslav Furtak, Margarita M. Ivanova, Erk Changsila, Floyd Wilks, and Marie N. Fidelia‐Lambert**: Critical revision of the manuscript for important intellectual content. **Ozlem Goker‐Alpan and Marjorie C. Gondré‐Lewis**: Funding acquisition. **Ozlem Goker‐Alpan andMarjorie C. Gondré‐Lewis**: Study supervision.

## DETAILS OF ETHICS APPROVAL

All procedures followed were in accordance with the ethical standards of the responsible committee on human experimentation (institutional and national) and with the Helsinki Declaration of 1975, as revised in 2000 (5). Informed consent was not obtained from the patients for being included in the study. The study was reviewed and exempted by Howard University Institutional Review Board (IRB‐14‐MED‐09) and the Western Institutional Review Board (NCT02120235) based on federal regulation 45 CFR 46 and associated guidance.

## Supporting information


**Supplemental table 1** Primers for *GBA*, *GAA*, and *GLA* genes PCR amplification.
