# Supplementary material for: Selective screening for lysosomal storage disorders in a large cohort of minorities of African descent shows high prevalence rates and novel variants
Source: JIMD Rep. 2021 Jan 27;59(1):60–8. doi: 10.1002/jmd2.12201 (PMC8100401; doi:10.1002/jmd2.12201)
Supplement: Supplementary file 1 — Supplemental table 1 Primers for GBA, GAA, and GLA genes PCR amplification. [file JMD2-59-60-s001.docx]

**Supplemental table 1**

| Exons | Forward Primer (5’ – 3’) | Reverse Primer (5’ – 3’) |
| --- | --- | --- |
| *GBA* (Gaucher disease) | | |
| Exon 1-2 | GCATCCTTGTTTTTGTTTAGTGG | CTCACCCCAAAGTTGGTCTC |
| Exon 3-5 | CAAGGGGTGAGGAATTTTGA | CACCACTGCACTCCTGTCTC |
| Exon 6-7 | CCAGCCTCCACAGGTTCCAACC | CAAGGCTGAAAGGCCCAGAA |
| Exon 6-7 | ACAGGTTCCAACCCAGGAGC | CTGATGGAGTGGGCAAGATTGACA |
| Exon 8 | AGGCTGTTCTCGAACTCCTG | GAGCCAGTCATTTGGATGCT |
| Exon 9 | AGTTGCATTCTTCCCGTCAC | TGATAGGCCTGGTATGGAATG |
| Exon 8-12 | GCCCAGCTGATTTTTCTATTTTTAG | ACTGGGGCTTACTGATCTTTTTCTA |
| *GAA* (Pompe disease) | | |
| Exon 1-2 | GGTTGATGTCTCAGAGCTGCTT | CTGCCCATCGTGTGAGAAA |
| Exon 3-7 | GGTGCTCTCTGGGTGCTCTC | CTGCACAGAGAAGGAGCCACT |
| Exon 8-11 | ACCCTCACCTTGACAGGTTTC | GCCCCAACCTTGTAGGACAG |
| Exon 12-14 | CTCTGCCTCATCCCAGAAAG | AGGCCCAAATGTTGTCTCACT |
| Exon 15-19 | CTTGAGCTCCAGAGAGCAGAAT | CTCCAGGTGACACATGCAAC |
| *GLA* (Fabry disease) | | |
| Exon 1 | CCAGTTGCCAGAGAAACAATAAC | GCAATGATCATGCATTAGTTTTAT |
| Exon 2 | ATACAAAGGATTGCAGGGAAAAT | GCGAATATGGTGAAACCTT |
| Exon 3-4 | GGCCGATGAAGACAGATTTTATT | TTTGTAGATAGGCAGGTGGGATA |
| Exon 3-7 | GCTAGGGATTTATGCAGATGTTG | AGTGAATGGAGAAAAAGGTGGAC |

**Supplemental table 1: Primers for *GBA*, *GAA*, and *GLA* genes PCR amplification.**
